# Investigation of MicroRNA and transcription factor mediated regulatory network for silicosis using systems biology approach

**DOI:** 10.1038/s41598-020-77636-4

**Published:** 2021-01-14

**Authors:** J. K. Choudhari, M. K. Verma, J. Choubey, B. P. Sahariah

**Affiliations:** 1 Chhattisgarh Swami Vivekanand Technical University, Bhilai, C.G 491107 India; 2 National Institute of Technology Raipur, Raipur, C.G 491020 India; 3 Raipur Institute of Technology, Raipur, C.G 492001 India

**Keywords:** Functional clustering, Gene ontology, Gene regulatory networks, Microarrays, Computational biology and bioinformatics, Systems biology, Diseases

## Abstract

Silicosis is a major health issue among workers exposed to crystalline silica. Genetic susceptibility has been implicated in silicosis. The present research demonstrates key regulatory targets and propagated network of gene/miRNA/transcription factor (TF) with interactions responsible for silicosis by integrating publicly available microarray data using a systems biology approach. Array quality is assessed with the Quality Metrics package of Bioconductor, limma package, and the network is constructed using Cytoscape. We observed and enlist 235 differentially expressed genes (DEGs) having up-regulation expression (85 nos) and down-regulation expression (150 nos.) in silicosis; and 24 TFs for the regulation of these DEGs entangled with thousands of miRNAs. Functional enrichment analysis of the DEGs enlighten that, the maximum number of DEGs are responsible for biological process viz, Rab proteins signal transduction (11 nos.) and Cellular Senescence (20 nos.), whereas IL-17 signaling pathway (16 nos.) and Signalling by Nuclear Receptors (14 nos.) etc. are Biological Pathway involving more DEGs. From the identified 1100 high target microRNA (miRNA)s involved in silicosis, 1055 miRNAs are found to relate with down-regulated genes and 847 miRNAs with up-regulated genes. The CDK19 gene (Up-regulated) is associated with 617 miRNAs whereas down-regulated gene ARID5B is regulated by as high as 747 high target miRNAs. In Prediction of Small-molecule signatures, maximum scoring small-molecule combinations for the DEGs have shown that CGP-60774 (with 20 combinations), alvocidib (with 15 combinations) and with AZD-7762 (24 combinations) with few other drugs having the high probability of success.

## Introduction

The health risks of silica (silicon dioxide, SiO_2_) primarily responsible for silicosis, one of the most frequent pneumoconiosis are highly documented as an occupational disease in mining workers and others with silica exposures^1–5^. Silicosis is characterized by injury to the alveolar cells preceded by an initial immune response and followed by expansion and activation of fibroblasts, and finally the deposition of the extracellular matrix (ECM). Exposure dose, both duration and concentration plays an important role in the development of silicosis types^6,7^. Exposure to crystalline silica (particles < 10 μm in diameter), amorphous silica (non-crystalline) and nano-silica (particles < 100 nm in diameter) exhibit a different effect on the development of silicosis, pulmonary fibrosis, and inflammation and cytotoxicity, respectively^8^. Silicosis is broadly categorized into three forms namely, Chronic silicosis, (develops due to exposure at low-moderate exposure for 10 or more years), Accelerated silicosis (develops from moderate to high-level exposure within 10 years) and Acute silicosis (results from intense exposure for a few weeks or to 5 years from the time of initial exposure).

Silicosis is illustrious irreversible lung fibrosis simultaneously coupled with many other diseases, such as pulmonary tuberculosis , lung cancer, renal failure, systemic sclerosis, rheumatoid arthritis, systemiclupus erythematosus, gastrointestinal problem, and autoimmune conditions, etc.^8,9^. Silica-induced lung damage occurs by several mechanisms including cell death by apoptosis, fibrosis and production of cytokines^10^. Due to the graveness of the element, International Agency for Research on Cancer (IARC) enlisted crystalline silica as a carcinogen^11^. There is possibility of no apparent symptoms at the initial stage, however, silicosis can continue even after cease of the silica exposure making the situation critical, non-curable, irreversible and uncontrolled immune processes^12^. Scientist and Medicine personnel recommend identification of workers at risk, prevention of further exposure to silica dust by job rotation and use of personal protective equipment, etc. to control the issue enhanced with early diagnosisand prediction^9,13^. Bandyopadhyay et al.^14^ stated that diagnostic challenges arise as silicosis shows resemblance in radiological and clinical overlap with pulmonary tuberculosis and neoplastic lesions.Therefore, it is necessary to investigate the complex molecular mechanisms that underlie the disease. Identification of differentially expressed genes (DEGs) in response to silica exposure and detail examination of the DEGs using the suitable statistical and computational approach may provide valuable information about the molecular mechanism(s) underlying the toxicity of crystalline silica^15^. The DEGs (upregulation/ downregulation) and/or their products, regulators, mostly transcription factors (TFs) and micro RNA (miRNA) following appropriate validation to recognize as a suitable biomarker(s) for silicosis is under investigation. A thorough investigation of possible molecular targets and mechanisms can make a way to achieve successful treatment options as well as prevention of potential adverse effects of silica exposure. The behavior of genes and genetic components due to silica exposure and toxicity are still rarely explained and are a vast area of scientific research^16^. Zhang et al.^17^. attempted to identify genome-wide aberrant DNA methylation profiling in lung tissues from silicosis patients using Illumina Human Methylation 450 K bead chip arrays. In the past, microarray-based transcriptomics studies have been successfully employed to gain insights into the molecular mechanisms underlying the toxicity of chemicals^7^ as well as to identify molecular markers for their toxicities^18^. Advances in high throughput gene expression profiling, such as transcriptomics, microarray analysis can enlighten in a better understanding of the effects of toxic agents in biological systems.

Wang et al.^19^ reported phagocytosis of SiO_2_ into the lung causes inflammatory on the cascade resulting in fibroblast proliferation and migration followed by fibrosis associated with monocyte chemotactic protein 1 and sustaine increase in p53 and PUMA protein levels. Wang et al.^19^ interpreted involvement of MAPK and PI3K pathways in the SiO_2_^−^ induced alteration of p53 and PUMA expression and to have possibility of link between SiO_2_-induced p53/PUMA expression in fibroblasts and cell migration on the basis of miRNAs that can interference with p53 and PUMA and prevent SiO_2_-induced fibroblast activation as well as migration. The study provide insight into the potential use of p53/PUMA in the development of novel therapeutic strategies for silicosis treatment. Therefore, in the current study we target to investigate the molecular mechanism underlying lung cell differentiation by identifying DEGs involved in silicosis, their expression (up/down-regulation), produce the possible network and gene ontology term analysis namely biological pathway and biological process for differentially expressed genes. We have also considered for identification of transcription factors (TFs) and microRNA (miRNA) associated with these DEGs for the regulation of the disease gene. A common network construction combining the relevant of TFs, miRNA, gene network, and small molecules for regulation of the disease progress is targeted.

## Methodology

### Microarray datasets

In this study, we have considered the work “Mechanisms of crystalline silica-induced pulmonary toxicity revealed by global gene expression profiling” and Dataset GSE30180 from the Gene Expression Omnibus (GEO) database (http://www.ncbi.nlm.nih.gov/geo)^16^. Datasets GSE30180 contains information regarding clinical tissues of human lung epithelial cells (A549 cells) considering 10 samples (5 control and 5 crystalline silica exposed to 800 ug/ml for 6 h using differential gene expression profile induced by silica conducted by National Institute for Occupational Safety and Health USA^16^). We have considered the available maximum concentration (800 ug/ml) from the study considering general worker’s shift exposure time period to have an idea at extreme conditions. Quality Control assessment of data quality is a major concern in microarray analysis. arrayQualityMetrics^20^ is from Bioconductor package that provides a report with diagnostic plots for one or two colour microarray data are used.

We have used arrayQualityMetrics for data quality assessment of microarray data and Quality Control is performed to identify potential low-quality arrays. The removal of low-quality arrays is desirable to avoid negative impact in downstream analysis procedures, by introducing invalid information and ultimately impairing statistical and biological significance. Array quality is assessed through the computation of commonly used statistical measures arrayQualityMetrics, an R package for quality control and quality assessment analysis that supports different types of microarrays in R. Here, we use an additional QC/QA that provides an HTML report with interactive plots. The instensity distribution of the arrarys of each box correspeonds to one array which indicated the over all quatity of each array with other corresponds are goods (Figure S1, S2).

### Screening of differentially expressed genes

To screen DEGs between control and crystalline silica exposed cell, differential expression analysis is conducted using Bioconductor. Bioconductor operates in R (a statistical computing environment) and is applied for genomic data analysis and comprehension. The normalized data is analysed for identification of DEGs by limma package 3.26.8 in R (following adjust = “fdr” sort by B”, number 250). The robust MultiArray average method^21^ is applied to perform background correction and data normalization using default parameters in the limma package^22^. Subsequently, a differential analysis between silica exposed and the no silica exposed is performed using the limma package^22^, a modified version of the standard t-test incorporating the Benjamini-Hochberg (BH) multiple hypotheses correction technique^23^. DEGs are defined as the false discovery rate (FDR) set as the cut‑off parameters to screen out 250 significant increases or decreases in gene expression levels^24^.

### Identification of transcription factor

IRegulon plugin^25^ in Cytoscape (version 3.8.0)^26^ is used to detect transcription factors using motif2TF and their optimal sets of direct targets for a set of genes Chip-seq data. The minimum identity between orthologous genes is 0.05%, while the maximum FDR on motif similarity is 0.001. The normalized enrichment score (NES) > 5 is considered as a threshold value for the selection of potential relationships. The NES for a given motif/track is computed as the Area Under the Recovery Curve (AUC) value of the motif/track minus the mean of all AUCs for all motifs (or tracks), and divided by the standard deviation of all AUCs. When the distribution of AUCs follows a normal distribution then the NES score is a z-score indicative of the significance. To maintain high accuracy of network inference large motif collections are collected from various species, and linking these to candidate human TFs via a *motif2TF* ^25^.

### Identification of miRNA (microRNA) for regulation Silicosis

To identify targets, regulators and interactions of the molecular factors included in the deferential gene expressed network, we search for gene-miRNA target and cross-validation using microRNA Data Integration Portal (mirDIP)^27^ in 30 database sources such as BCmicrO, BiTargeting, CoMeTa, Cupid, DIANA, ElMMo3, GenMir +  + , MAMI, MBStar, MirAncesTar, MirMAP, MirSNP, MirTar, Mirza-G, MultiMiTar, PACCMIT, PITA, PicTar, RNA22, RNAhybrid, RepTar, TargetRank, TargetScan, TargetSpy, miRDB, miRTar2GO, miRcode, microrna.org, mirCoX and miRbase for regulating the expressions using both unidirectional and bidirectional search method. All the database considered in the present study is from authenticated and publically available sites^27^.

### Functional enrichments analysis

ClueGO and CluePedia^28^ plug-in of Cytoscape is used for functional enrichment analysis. ClueGO plug-in translates functionally grouped Gene Ontology (GO) and pathway annotation networks with a hypergeometric test along with the kappa coefficient^28^ of pathways as well as functional correlations among pathways. ClueGO provides enrichment scores for selected gene sets against the user-provided gene list. CluePedia^29^ finds new markers that are potentially related with pathways and extend ClueGO functionality with other biological data deriving screened results. Default parameters are used, and only GO terms with *P* < 0.05 are selected in ClueGO with a Benjamini–Hochberg correction and a kappa score of 0.5 (medium).

### Network construction and analysis of clusters

Finally, regulatory networks are constructed for silicosis by merging selected DEGs and TFs-DEGs pairs using Cytoscape. Thereafter, the Molecular Complex Detection (MCODE) plug-in^23^ is used to screen Clusters of hub genes from the network with degree cut-off = 10, haircut on, node score cut-off = 0.2, k-core = 2, and max. depth = 100.

### Prediction of small-molecule signatures considering DEGs of silicosis

L1000CDS^2^ web tool is used for prediction of the potential small-molecule signature that matches user input signature genes expressions based on characteristic direction method in the underlying dataset. It is an ultra-fast LINCS L1000 Characteristic Direction Search Engine for prediction the potential small-molecule signature^30^ and DEGs are pasted into up/down text box and the top 50 signatures are considered.

## Results and discussion

### Identification of differentially expressed genes

The considered dataset is applied for significant DEGs identification by Bioconductor and 235 DEGs are found to be significantly associated with silicosis disease on the basis of the considered criteria. Table 1a, b enlist total of 235 DEGs, where down-regulated genes and up-regulated genes are 150 and 85 nos, respectively. On the basis of important pathway analysis and GO term Sellamathu et al.^16^ identified 60 DEGs for crystalline silica exposure in their study.

**Table 1:** DEGs gene in Silicosis.

Sl. no	Symbol	logFC	*P* value	Sl. no	Symbol	logFC	*P* value
**(a) Down-expression**							
1	LTB	− 0.3411	3.38E−07	76	MYC	− 1.1121	3.18E−08
2	CDCP1	− 0.3691	1.25E−07	77	EIF1	− 1.1267	2.77E−07
3	ABL2	− 0.4115	1.53E−07	78	CITED4	− 1.133	4.62E−10
4	EEA1	− 0.415	1.07E−07	79	C16orf72	− 1.1369	2.89E−10
5	SERPINB8	− 0.4776	6.51E−08	80	PMP22	− 1.1442	2.10E−08
6	ZC3H12A	− 0.4998	8.23E−08	81	CDKN1A	− 1.215	3.91E−08
7	ABTB2	− 0.5189	9.46E−08	82	FOSL1	− 1.2205	2.31E−09
8	KLF10	− 0.5365	9.83E−08	83	TRIM8	− 1.2315	2.95E−08
9	IL1B	− 0.5375	1.99E−07	84	DDIT4	− 1.2323	1.38E−08
10	ELF3	− 0.5526	2.55E−07	85	AEN	− 1.2672	2.30E−08
11	UBAP1	− 0.5561	9.97E−08	86	MCL1	− 1.2687	1.50E−08
12	SLC25A25	− 0.5606	1.19E−07	87	SERPINE1	− 1.3011	4.86E−08
13	TP53BP2	− 0.5608	6.30E−08	88	SOD2	− 1.3201	1.41E−11
14	NAV3	− 0.569	4.66E−08	89	C3orf52	− 1.3231	6.38E−10
15	KLHL21	− 0.572	5.85E−09	90	F2RL1	− 1.3284	3.64E−08
16	ZBTB20	− 0.5817	8.21E−08	91	NRG1	− 1.3386	1.43E−08
17	CD274	− 0.5835	7.31E−08	92	FRMD6	− 1.3479	3.62E−10
18	SH3KBP1	− 0.5872	3.49E−08	93	NOCT	− 1.3675	3.50E−07
19	TEX10	− 0.5877	4.32E−08	94	CLCF1	− 1.374	8.16E−10
20	TUBB2B	− 0.5894	1.71E−07	95	BTG1	− 1.3812	2.45E−07
21	HIST1H4H	− 0.5987	9.47E−08	96	IL1A	− 1.388	6.36E−11
22	EREG	− 0.6072	1.15E−08	97	ERRFI1	− 1.4065	1.00E−08
23	HIST1H4B	− 0.6251	1.14E−09	98	HIST2H2AA3	− 1.4396	2.32E−08
24	HIST1H2BD	− 0.637	5.37E−08	99	PDK4	− 1.4875	3.06E−07
25	SPRY4	− 0.6452	7.90E−09	100	TIPARP	− 1.5105	9.14E−11
26	MNT	− 0.6567	4.76E−09	101	EFNA1	− 1.5507	1.79E−08
27	ZNF787	− 0.6587	3.67E−08	102	HES1	− 1.5686	8.80E−09
28	TNFRSF10A	− 0.6637	2.62E−07	103	DUSP1	− 1.5838	3.65E−11
29	VPS37B	− 0.666	1.74E−08	104	BHLHE40	− 1.6055	1.92E−09
30	PPP3R1	− 0.6884	4.75E−08	105	TRIB3	− 1.6163	3.24E−09
31	PPARG	− 0.7212	1.64E−07	106	CSRNP1	− 1.6418	2.52E−10
32	SNAI2	− 0.7478	2.92E−07	107	JUND	− 1.707	3.89E−11
33	HIST1H2BK	− 0.7562	2.39E−07	108	NFKBIZ	− 1.7252	7.40E−10
34	BRD2	− 0.7742	5.13E−08	109	ZFP36L1	− 1.733	2.30E−07
35	SLC25A37	− 0.7751	7.63E−08	110	CXCL5	− 1.7693	4.87E−11
36	CITED2	− 0.7784	1.58E−08	111	HBEGF	− 1.7961	1.48E−12
37	RASD1	− 0.7834	5.28E−10	112	SMOX	− 1.8185	3.67E−09
38	EPAS1	− 0.7965	1.70E−08	113	SOX9	− 1.8386	3.29E−10
39	RELB	− 0.7966	2.62E−09	114	ETS1	− 1.8392	1.59E−10
40	HIST2H2AC	− 0.8065	3.30E−08	115	CEBPB	− 1.8569	4.29E−10
41	MYEOV	− 0.8132	1.55E−08	116	ARID5B	− 1.8615	4.60E−10
42	VGF	− 0.8144	1.93E−08	117	RND3	− 1.8847	9.85E−09
43	ZNF296	− 0.8163	2.07E−07	118	CSF2	− 1.9288	1.13E−11
44	PHLDA2	− 0.8249	2.43E−09	119	GADD45A	− 1.93	2.46E−10
45	ZNF34	− 0.8412	1.81E−07	120	MMP10	− 1.9343	2.46E−11
46	SDC4	− 0.8467	2.17E−08	121	TRIB1	− 1.9631	5.73E−10
47	NPC1	− 0.8749	2.46E−08	122	SOWAHC	− 2.0047	2.17E−09
48	FOXQ1	− 0.8766	1.73E−07	123	DDIT3	− 2.0533	2.47E−11
49	SEMA4B	− 0.9049	4.29E−08	124	CCL20	− 2.055	4.35E−09
50	TSC22D1	− 0.9076	1.84E−08	125	NFKBIA	− 2.1639	3.84E−12
51	ODC1	− 0.9077	3.40E−10	126	SERTAD1	− 2.1667	5.24E−11
52	ID3	− 0.9131	1.81E−07	127	FST	− 2.2029	1.82E−11
53	CCNL1	− 0.9141	3.46E−08	128	IL11	− 2.2994	5.24E−11
54	HAS2	− 0.9215	7.31E−10	129	NR4A2	− 2.3141	9.86E−11
55	ITPRIP	− 0.922	1.23E−08	130	PHLDA1	− 2.3232	5.65E−10
56	HES4	− 0.9358	1.66E−10	131	TNFAIP3	− 2.3238	6.14E−12
57	KCNF1	− 0.95	2.37E−07	132	TMEM158	− 2.3369	7.44E−12
58	PIM1	− 0.9635	1.97E−07	133	STC1	− 2.4343	5.40E−14
59	CREBRF	− 0.9638	5.34E−08	134	GEM	− 2.7581	1.41E−10
60	FOXD1	− 0.971	1.84E−08	135	KLF6	− 2.7745	4.91E−12
61	TICAM1	− 0.9763	2.72E−10	136	IRAK2	− 2.8376	5.47E−11
62	DDX10	− 0.9801	2.20E−09	137	IER3	− 2.9841	8.31E−13
63	ZNF143	− 0.9831	2.08E−07	138	DUSP5	− 3.0033	4.70E−12
64	TSC22D2	− 0.9901	1.97E−08	139	CXCL2	− 3.0447	4.31E−13
65	CLK1	− 0.9959	7.49E−08	140	BIRC3	− 3.0477	4.14E−14
66	EHD1	− 1.0058	1.26E−09	141	IL6	− 3.09	4.80E−14
67	RYBP	− 1.0184	2.31E−08	142	GDF15	− 3.2752	5.89E−14
68	STC2	− 1.0209	2.01E−07	143	JUN	− 3.4108	8.15E−13
69	TMEM156	− 1.037	4.80E−08	144	ZFP36	− 3.4123	6.61E−11
70	FBXO32	− 1.0608	5.93E−09	145	PPP1R15A	− 3.4502	5.73E−14
71	PTHLH	− 1.0665	2.10E−10	146	PTGS2	− 3.9915	1.10E−12
72	AGO2	− 1.0675	6.71E−09	147	FOS	− 4.3462	1.87E−12
73	ISG20	− 1.0961	3.05E−10	148	FOSB	− 5.118	7.37E−15
74	PLAUR	− 1.098	3.53E−10	149	EGR1	− 5.1837	7.67E−15
75	SERTAD2	− 1.104	2.80E−09	150	CXCL8	− 5.3785	7.60E−17
**(b) Up-expression**							
1	MAT2A	1.9868	8.29E−09	44	MUM1	0.6787	7.56E−08
2	TNS3	1.4299	4.96E−08	45	AFAP1L2	0.6772	3.33E−10
3	FZD2	1.3399	3.41E−08	46	RSPRY1	0.6733	7.20E−08
4	BAAT	1.173	2.88E−10	47	FAM120B	0.666	1.13E−07
5	SRSF5	1.1311	2.43E−08	48	IDH1	0.6532	3.22E−07
6	KIF20A	1.1296	8.54E−10	49	STK36	0.6508	1.52E−07
7	MIR503HG	1.0687	2.56E−08	50	OIP5	0.6505	6.00E−08
8	FAM83D	1.0517	2.22E−08	51	SUOX	0.6391	1.59E−08
9	PDXK	1.0353	5.96E−08	52	PHLDB1	0.6384	8.64E−08
10	FAM217B	1.0242	9.44E−09	53	AP3M2	0.634	2.75E−08
11	MED20	1.0059	2.15E−08	54	CKAP2	0.6166	4.08E−08
12	CENPF	0.9756	1.14E−08	55	C17orf58	0.6126	6.55E−08
13	TMEM203	0.9476	3.90E−08	56	LGR4	0.6104	6.92E−08
14	CPSF4	0.9226	1.37E−08	57	RNFT2	0.6083	1.46E−07
15	MSRB1	0.9176	2.00E−07	58	MCEE	0.5906	8.08E−08
16	EPDR1	0.9021	5.20E−08	59	ZNF30	0.5901	2.43E−07
17	GEMIN6	0.8992	1.65E−07	60	PHF21A	0.5889	2.24E−07
18	HSPA2	0.8958	3.12E−08	61	FAM64A	0.5868	1.58E−07
19	PDE7B	0.8926	1.33E−08	62	NREP	0.5857	1.20E−07
20	LACTB	0.8845	1.37E−08	63	OGT	0.5782	1.18E−07
21	ANG	0.8734	3.98E−08	64	FANCL	0.5766	1.19E−07
22	TMEM2	0.8729	5.82E−09	65	ZBED8	0.561	3.45E−08
23	MALSU1	0.8366	1.34E−07	66	KLHL12	0.5563	8.06E−09
24	RAB40B	0.8208	1.70E−07	67	GMCL1	0.5349	2.89E−07
25	IMP3	0.8158	5.93E−08	68	CCDC117	0.5166	3.28E−07
26	SKP2	0.7971	3.97E−09	69	ANKRA2	0.5163	1.73E−07
27	GID8	0.7961	1.26E−07	70	CBX2	0.5119	5.66E−08
28	GSPT2	0.7938	8.55E−08	71	CCDC25	0.5029	2.14E−07
29	HOXC8	0.7813	1.93E−07	72	TIA1	0.5002	8.52E−08
30	TOP2A	0.7797	1.84E−07	73	AGGF1	0.4931	2.06E−07
31	DCBLD1	0.7778	6.79E−08	74	NUPL2	0.4768	1.63E−07
32	CDCA8	0.7776	6.30E−08	75	CCDC34	0.4618	1.46E−07
33	CXXC5	0.7511	1.01E−07	76	THNSL1	0.4595	1.86E−07
34	UNC50	0.7461	2.13E−07	77	SNUPN	0.4576	1.52E−07
35	OARD1	0.7395	3.17E−07	78	MAGEE1	0.4561	1.55E−07
36	POC1A	0.7372	7.79E−08	79	ASB13	0.4215	3.36E−07
37	AURKA	0.7346	3.10E−07	80	PXYLP1	0.4214	7.53E−08
38	HNRNPA0	0.7228	2.46E−09	81	AXIN2	0.4176	1.65E−07
39	RBM4B	0.7161	1.61E−07	82	RNASE4	0.4174	3.70E−08
40	C1orf131	0.7144	3.04E−08	83	CDK19	0.4174	1.95E−07
41	TSEN2	0.7092	2.24E−07	84	ZNF17	0.4124	3.44E−07
42	ZNF512	0.7006	2.02E−07	85	CDH1	0.3405	2.50E−07
43	SLC35E3	0.6925	9.92E−08				

### Identification of transcription factors (TFs) for differentially expressed genes

Adopting the iRegulon plugin in Cytoscape, 24 TFs are identified from publicly available database signatures/genesets (GeneSigDB, Ganesh clusters or/and MSigDB) that are significantly associated with the DEGs involved in the silicosis disease. It is found that 20 TFs influence both up and down-expressed genes whereas four TFs are solely involved in controlling only the down-regulated genes (Table 2).

**Table 2:** Identified TFs for regulation DEGs genes in Silicosis.

Sl. no	TF	Target gene with expressions	
1	CHD1	Up	A34A1:A25
		Down	CEBPB, PPP1R15A, DUSP1, DUSP5, FOSB, FOSL1, MCL1, KLF10, JUN, CDKN1A, JUND, ATF3, NR4A2, SOD2, FOS, SOX9, RELB, ERRFI1, SNAI2, TP53, SERPINE1, EGR1, STC2, CSRNP1IL6, RND3, ETS1, EIF1, PIM1, DDIT3, TP53BP2, ELF3, TSC22D1, TSC22D2, KLHL21
2	ATF4	Up	NA
		Down	FOSB, ATF3, CDKN1A, JUN, FOSB, ERRFI1, EIF1, DDIT4, CEBPB, TRIB3, CCL20, HAS2, PPARG, DDIT3, STC2, IL1A
3	BACH1	Up	BAAT, OGT, PHF21A, CXXC5, EPDR1
		Down	FOSB, FOSL1, DUSP1, DUSP5, NR4A2, CDKN1A, IL6, CEBPB, SOD2, PPARG, ATF3, SOX9, HAS2, RELB, ERRFI1, RND3, ETS1, PIM1, IL1A, DDIT3, STC2, SERPINE1, TSC22D1, TSC22D2, TRIB3, EEA1, CSF2, BIRC3, PTGS2, TNFAIP3, CITED2, CSRNP1, F2RL1, KLHL21, EREG, CXCL2, SERTAD1, PTHLH, HBEGF
4	CEBPG	Up	CXXC5, OGT, CENPF, GEMIN6, PHF21A, SLC35E3, BAAT, CDK19, EPDR1, FAM83D, MED20, CBX2
		Down	ATF3, CDKN1A, ERRFI1, RND3, ETS1, DDIT3, DDIT4, SNAI2, SERPINE1, EGR1, STC2, TSC22D2,TSC22D1, TRIB3, HAS2, IL1A, EEA1, CSF2, PTGS2, TNFAIP3, CXCL2, PPARG, CSRNP1, KLHL21, EREG, CEBPB, PPP1R15A, SOX9, PPP3R1, NR4A2, EIF1, DUSP1, FOS, FOSB, FOSL1, MCL1, KLF10, JUN, JUND, IL6, NR4A2 RELB,, PTHLH, NFKBIA, BIRC3, HIST1H2BD, CITED2
5	KAT2A	Up	TMEM203
		Down	FOS, FOSB, FOSL1, DUSP5, MCL1, CDKN1A, KLF10, NR4A2, JUN, SOD2, JUND, ERRFI1, ATF3, EIF1, PIM1, DDIT4, EGR1, TSC22D1, HIST1H2BD
6	PAX3	Up	NA
		Down	FOS, FOSB, NR4A2, FOSL1, RELB, PPARG, KLF10, ERRFI1, EIF1, EGR1, RND3, TSC22D2, HAS2, PTHLH, PPP1R15A, TP53BP2, ATF3, FOSB, CDKN1A, IL6
7	FOXF1	Up	BAAT, PHF21A, CXXC5, CDH1, CDK19
		Down	FOSB, FOSL1, DUSP5, KLF10, IL6, CDKN1A, PPP1R15A, KLF10, JUND, NR4A2, SOX9, ERRFI1, TNF, RND3, ETS1, EIF1, DDIT4, ELF3, SERPINE1, EGR1, STC2, TRIB3, CEBPB, HAS2, EEA1, CSF2, BIRC3, CITED2, F2RL1, KLHL21, CXCL2, PTHLH, NFKBIA, HBEGF, HIST1H2BD, RELB, PPP3R1
8	CREB3	Up	CXXC5, CDK19, CENPF
		Down	DUSP1, FOS, FOSB, DUSP5, JUND, NR4A2, CDKN1A, JUN, ATF3, EIF1, IL6, SOX9, HAS2, EGR1, PPP1R15A, RELB, TNFAIP3, CITED2, KLHL21, PTHLH, STC2, HIST1H2BD, ERRFI1, IL1A, TSC22D2, CEBPB, PPARG, PPP3R1, HBEGF
9	POLR2A	Up	NA
		Down	FOS, FOSB, MCL1, NR4A2, JUND, PPP1R15A, DUSP1, JUN, EIF1, DDIT3, EREG, HIST1H2BD, EGR1
10	HOMEZ	Up	CDK19, CENPF, CXXC5, OGT
		Down	ATF3, NR4A2, ETS1, PIM1, CDKN1A, PPARG, PPP3R1, TP53BP2, CSRNP1
11	CEBPB	Up	PHF21A, OGT
		Down	KLF10, NR4A2, ATF3, SOD2, PPP1R15A, CDKN1A, DDIT4, TRIB3, HAS2, IL1A, EREG, CXCL2, NFKBIA, HIST1H2BD, PPARG
12	ZNF513	Up	PHF21A, CXXC5
		Down	FOS, FOSB, NR4A2, JUND, DDIT4, EGR1, TSC22D1, HAS2, TNFAIP3, ATF3, F2RL1, CDKN1A, TSC22D2, ERRFI1, PIM1, HIST1H2BD, CITED2, PPARG
13	E2F1	Up	PHF21A, CXXC5
		Down	ATF3, DUSP5, NR4A2, SOX9, IL6, ERRFI1, RND3, ETS1, PIM1, TSC22D1, HAS2, BIRC3, PTGS2, TNFAIP3, F2RL1, TSC22D2
14	EGR2	Up	RELA,
		Down	FOS, NR4A2, CDKN1A, ETS1, PIM1, EGR1, HAS2, PTHLH
15	FOXM1	Up	FAM83D, CENPF, CXXC5, KIF20A, TOP2A, RNFT2, ASB13, AURKA, OIP5, CDK19, CDH1, CDCA8
		Down	FOS, KLF10, SOX9, PIM1, ELF3, STC2, TSC22D2, CITED2, EREG, HIST1H2BD, C3orf52
16	ZNF683	Up	PHF21A, CXXC5
		Down	FOS, JUND, NR4A2, CDKN1A, DDIT4, HAS2, SERTAD1, PTHLH, ERRFI1, STC2, ATF3
17	RELA	Up	OGT, PHF21A, EPDR1, CXXC5, KIF20A, CBX2, GEMIN
		Down	FOSB, DUSP5, NR4A2, CDKN1A, IL6, ATF3, CSRNP1, JUND, SOX9, TP53, CSF2, HAS2, PPARG, PTHLH, DDIT4, BIRC3, TNFAIP3, CITED2PIM1, ETS1, STC2, SERPINE1, EGR1, TSC22D1, CCL20, CENPF, ERRFI1, TNF, RND3, EIF1, F2RL1, EREG, NFKBIA, HBEGF, HIST1H2BD, PPP3R1
18	MEF2D	Up	CXXC5, BAAT, PHF21A, OGT
		Down	ATF3, JUND, PPP1R15A, FOSB, NR4A2, CEBPB, SOX9, TNF, CDKN1A, HAS2, ELF3, EGR1, PTHLH, RELB, ERRFI1, EIF1, DDIT4, TRIB3, SERPINE1
19	FOXP2	Up	PHF21A, CDK19, FAM83D
		Down	FOSB, FOSL1, DUSP5, KLF10, JUND, ATF3, DUSP1, CDKN1A, CSRNP1, JUN, F2RL1, CITED2, SOD2, ERRFI1, EEA1, ETS1, EIF1, PIM1, DDIT3, STC2, EGR1, DDIT4, TP53BP2, PPP3R1, TSC22D2, CEBPB, TRIB3, KLHL21, SERTAD1, HBEGF
20	PSMC2	Up	CXXC5
		Down	KLF10, NR4A2, CDKN1A, EIF1, ETS1, NFKBIA
21	JUND	Up	NA
		Down	FOSL1, NR4A2, CSRNP1, SOX9, PPARG, ERRFI1, DDIT4, EGR1, TSC22D1, DDIT3, CITED2, F2RL1, EREG, PTHLH, HIST1H2BD
22	ESR1	Up	FAM83D
		Down	ATF3, CDKN1A, CSRNP1, ETS1, CCL20, NFKBIA,
23	TBPL2	Up	PHF21A, CDH1, CXXC5, BAAT
		Down	ATF3, FOSB, DUSP5, NR4A2, JUND, FOS, SOX9, ERRFI1, EGR1, STC2, EIF1, TSC22D1, CDKN1A, DDIT4, KLHL21, PTHLH, TNF
24	RDBP	Up	FAM83D, OIP5
		Down	ATF3, FOS, FOSL1, JUN, DUSP1, EIF1, DDIT3, DDIT4, EGR1, CITED2, HIST1H2BD

### Identification of miRNA for DEGs from database-driven expansion of the network

Using CyTargetLinker^31^ authors observed initially only 2716 miRNAs responsible for silicosis affected gene regulation from databases as mentioned in the methodology. Therefore, the authors further explored a few other databases as mentioned in the methodology (public databases around 30 nos). We applied mirDIP and observed a total of 2586 miRNAs associated with targeted DEGs responsible for silicosis and categorized as “very high” (top 1%) and “high” (top 5% (excluding top 1%))^27^. A total of 1105 miRNA is categorized as very high where 846 miRNAs influence upregulated genes, 1055 miRNAs regulate down-regulated genes and 801 miRNAs regulates both (Supplementary file 1). For example, among identified miRNA, miRNA- 29 influences epithelial-mesenchymal transition^32^, and miRNA-489 can repress its target genes MyD88 and Smad3 responsible for silicosis^33^. Chen et al.^34^ reported the involvement of IL-10-producing B cells in the development of silica-induced lung inflammation and fibrosis of mice and many such in the list. Yang et al.^35^ observed significant genetic heterogeneity involved in the origin and development of silicosis from research data and recommended relevant miRNAs as biomarkers having a role in regulating pulmonary fibrosis. The research group reported differential miRNAs in leukocytes as up-regulated (18 nos.) and down-regulated (20 nos.) during silicosis, compared with the control group miR-19a in peripheral blood leukocyte^35^. Faxuanet al.^36^ observed 39 differential expression miRNAs (14 up-regulated and 25 down-regulated in silicosis sample) between silicosis and normal lung tissues. Zhang et al.^37^ performed genome-wide miRNAs expression profiling in BALF cell fraction of 3 silicosis observation stages simultaneously with 6 silicosis patients. Among the identified 110 dysregulated miRNAs having down-regulation trend, 23 miRNAs abundantly expressed in stage I and Stage II silicosis suggesting different stages of silicosis are associated with distinct changes in miRNAs expression.

### Regulatory network construction

We identified targets, regulators and integrators of the molecular factors included in the DEG Network. Gene expressed network is a node and edge interaction between gene–gene or gene-miRNA and regulatory transcription factors. Precisely, we searched for gene regulatory interaction network as a) TF-target gene interactions and b) miRNA–gene interactions. We merged all the extracted interactions with the gene expressed Network using the same annotation principles as above.

#### TFs-DEGs network analysis

**Figure 1:**
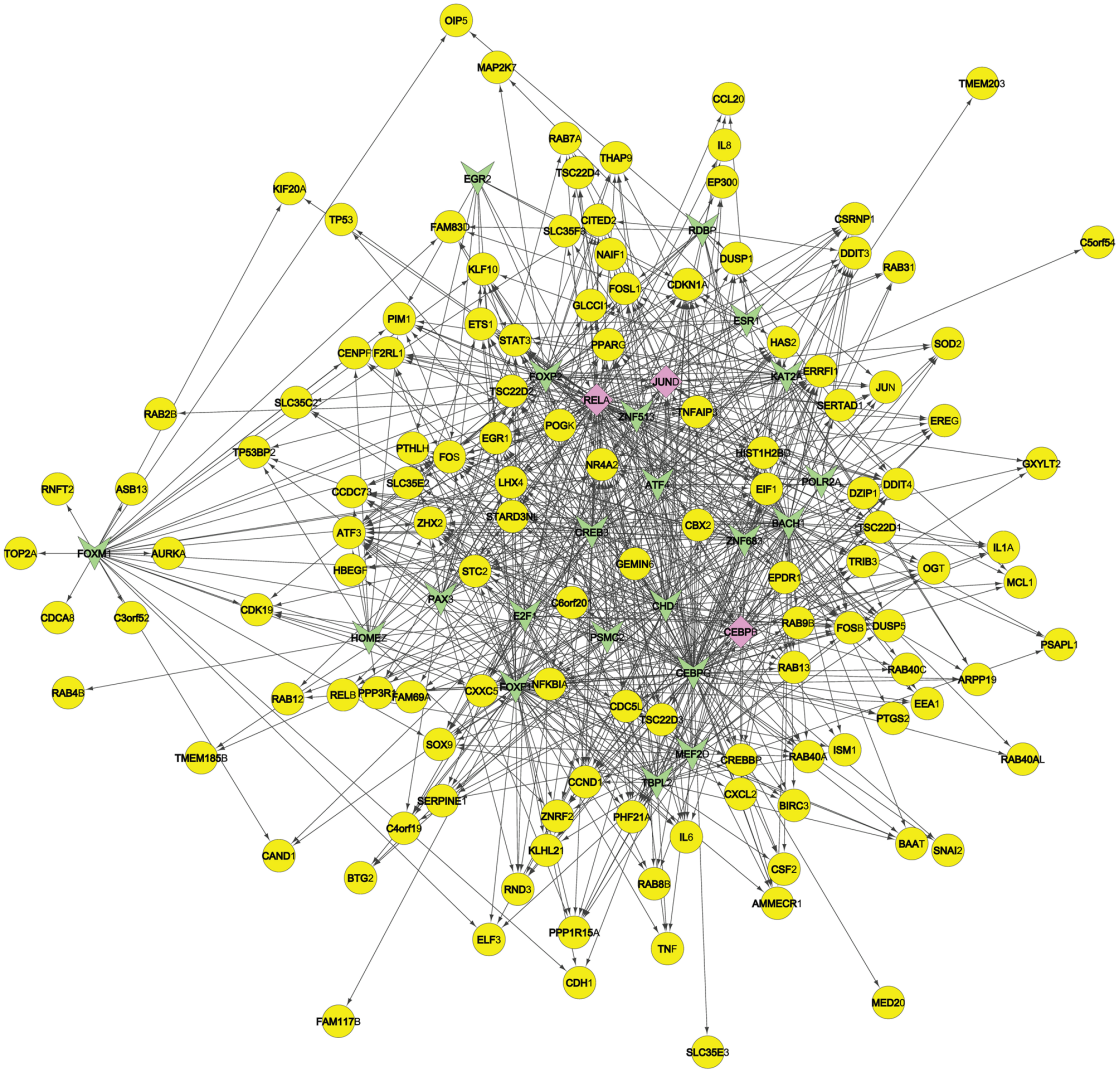
The gene-transcription factor regulation network. Notes: a round node represents a gene (yellow node) with differencial expresstion, a triangle nodes (green node) represents the TFs, and a rectangular rectangular nodes (pink node) represent the TFs that plays a role as regulator and regulated.

The interaction network is derived using plugin Cytoscape with 147 Nodes and 769 edges (Fig. 1). The blue nodes indicate here are the genes namely, RELA, JUND, and CEBPB which act simultaneously as TF, so function as both regulator and regulated gene.

The most interacting TFs are CHD1, CEBPG, FOXF1, CREB3, RELA, and FOXP2, etc. as given in Table 2. The TF CHD1 is a gene similar to various other genes that act as TF and can regulate the activity of certain genes providing instructions to make epithelialcadherin or E-cadherin (a protein) having influence in cell adhesion, the transmission of chemical signals within cells, control of cell maturation and movement^38^. ATF4 TF is known to regulate memory, metabolism, and adaptation of cells to stress factors such as anoxic insult, endoplasmic reticulum stress, and oxidative stress^39^. In normal bronchial epithelial cells, CEBPG TF correlates with antioxidant and DNA to repair genes which is absent for individuals with bronchogenic carcinoma^40^. Expression of KAT2A(Tyr645Ala), reduces gene expression and inhibits tumor cell proliferation as well as tumor growth^41^. CREB3 influences leukocyte migration, tumor suppression, and endoplasmic reticulum stress-associated protein degradation^42,43^. PAX3 plays critical roles during fetal development as well as neural crest^44,45^. Mutations in paired boxgene 3 are associated with Waardenburg syndrome, craniofacial-deafness-handsyndrome, and alveolar rhabdomyosarcoma^46^. FOXF1 promote slung regeneration after partial pneumonectomy and involved in murine vasculogenesis, lung, and foregut development. Mice with reduced levels of pulmonary FOXF1 may face death due to pulmonary hemorrhages with deficient alveolarization and vasculogenesis^47,48^. POLR2A geneis associated with poor overall and disease-free survival of patients with early-stage non-small cell lung cancer^49^. FOXP2 is responsible for defective postnatal lung alveolarization resulting in postnatal lethality in mice. T1 alpha, a lungalveolar epithelial type 1 cell-restricted gene crucial for lung developmentand function, is a direct target of FOXP2 and FOXP0. Both FOXP2 and FOXP1 are crucial regulators of lung and oesophageal development^50^. E2F1significantly influences S phase progression and apoptosis as well FOXM1 expression, cell survival, epirubicin resistance^51^ and its low level in primary lung adenocarcinoma may lead to cancer^52^. In small cell lung cancer, activation of oncogene EZH2 is often triggered by genomic deregulation of the E2F/Rb pathway^53^. Alveolar macrophages from RELA deficient animals are significantly less capable to involve in the canonical NF-κB pathway (a prototypical immune transcription pathway) and stimulate epithelial cells^54^.

#### miRNA regulatory network analysis

**Figure 2:**
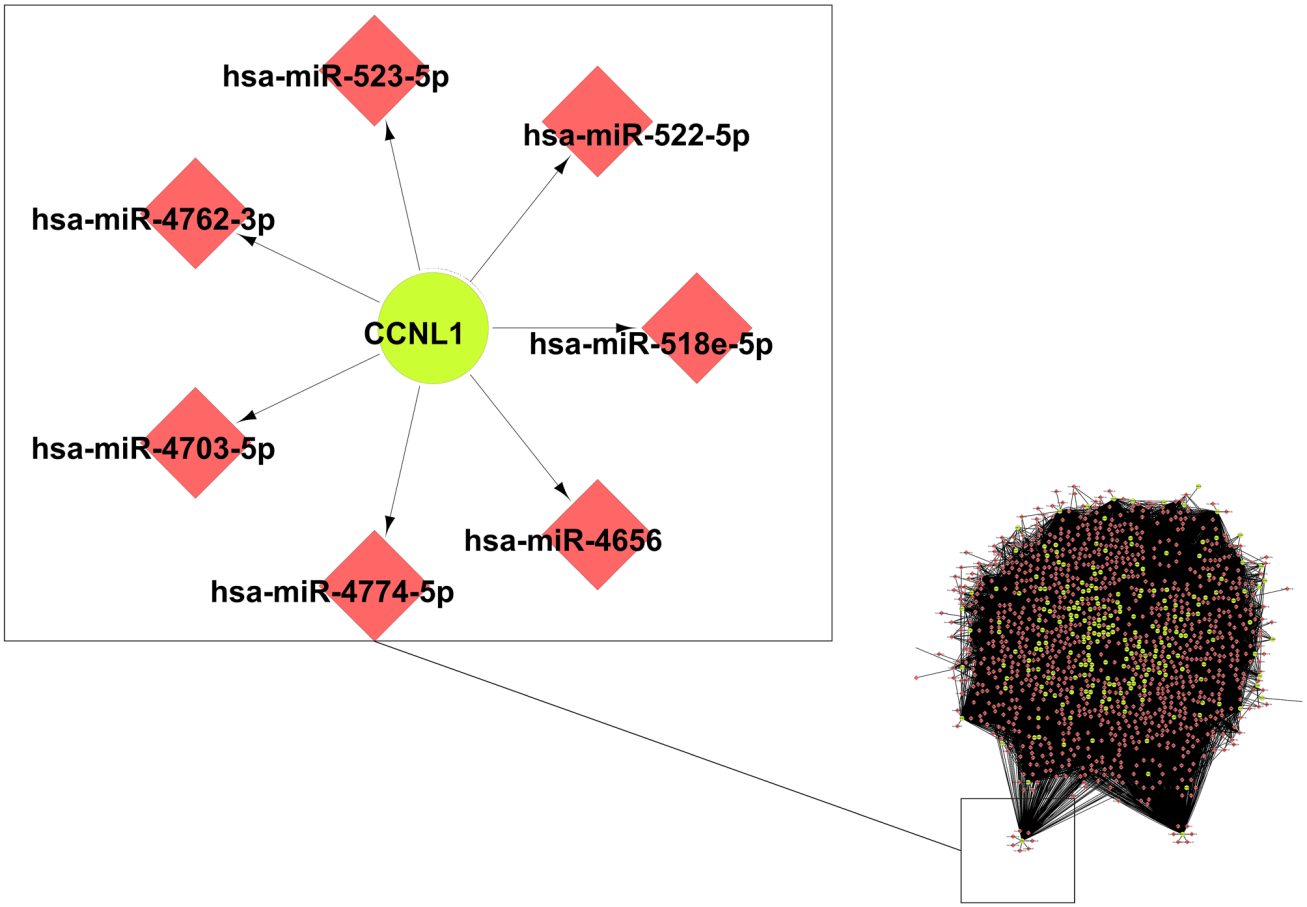
Gene–miRNA interactions network; index red node (miRNA); grey node (gene).

In present study, from the dataset GEO30180 we enlisted significant DEGs and investigated associated miRNAs using CyTargetLinker and in-silico validation for miRNA through cross validation with the multiple database mirDIP is carrird out for creation of Gene-TFs-miRNAs regulatory network. We have chosen the miRNA and mRNA from microRNA Data Integration Portal (mirDIP) web tools. In present manuscript, authors tried to identify the significant DEs, regulatory TFs and relevant miRNAs and predict the small molecules for drug combination of silicosis. Interaction network for genes and miRNAs is derived with 2461 Nodes and 13,343 edges as given in Fig. 2 using a plug-in CyTranslinker. An enlarged part is showing GPRY gene with its regulating miRNAs as an example. A grand total of 1100 number of very high target miRNA is identified from the considered database mirDIP using score class “very high”. For many upregulated genes, miRNA (numbers are given in parenthesis) is associated in several hundred; for example, CDK19 (617), OGT (552), LGR4 (426), MAT2A (409),NREP (408), TIA1 (387), and TMEM2 (202).

Similarly, for down-regulated genes, a grand total of 1055 very high target miRNA is identified to be associated with theconsidered database mirDIP using score class “very high”. For example, RYBP (899), SERTAD2 (666), CREBRF (675), NAV3 (629), PPP3R1 (560), TSC22D2 (555), STC1 (523), MCL1 (463), EEA1(414), and ZFP36L1(387).

#### Integrating TF and miRNA regulatory networks

TFs induce micro RNAs (miRNAs) transcription and miRNAs influence mRNA translation as well as transcript degradation for the regulation of gene expression and all these results in complex relationships feedback or feed-forward loops^54–56^.

**Figure 3:**
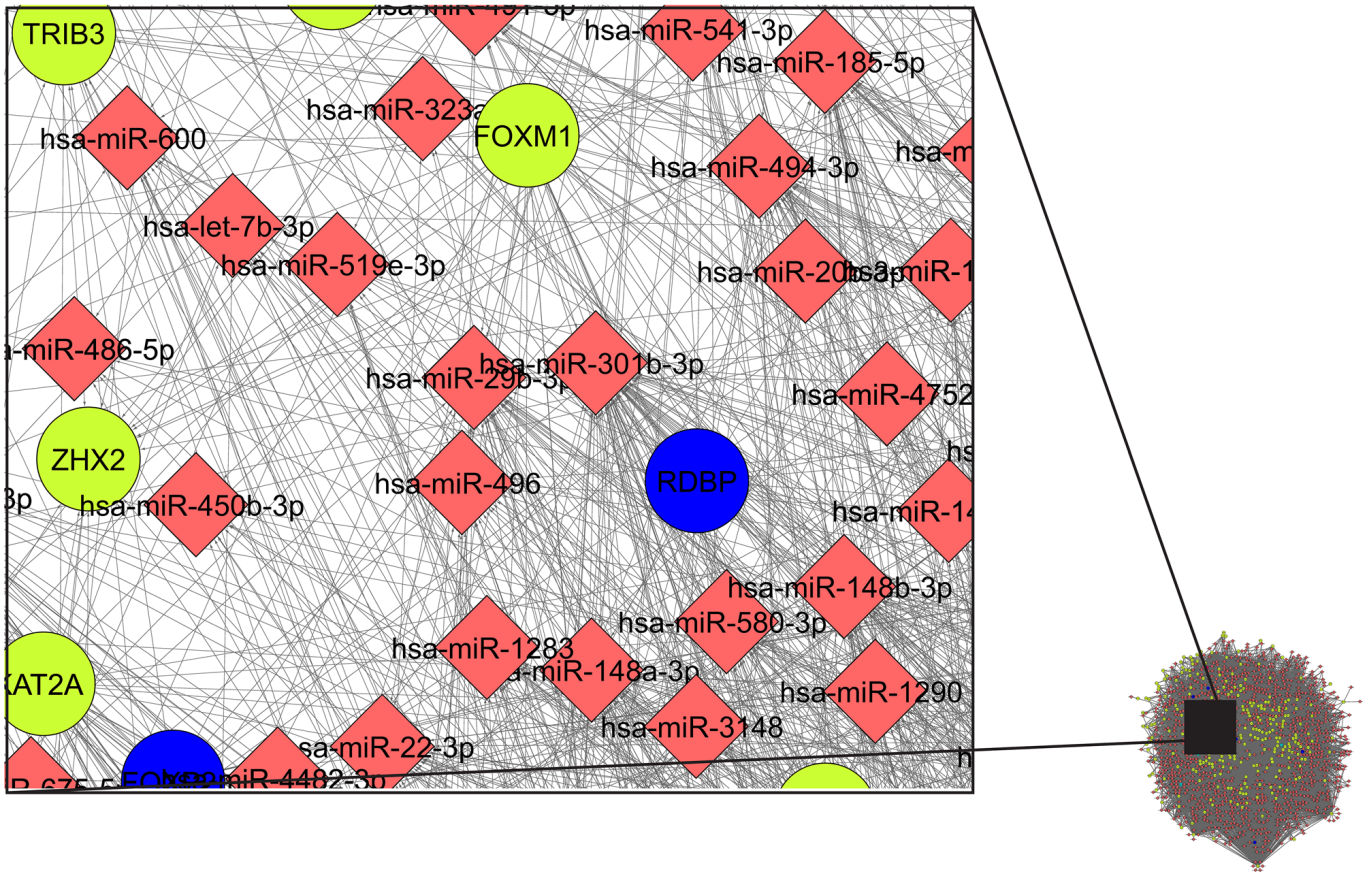
Gene–TF-miRNA regulatory network for Silicosis disease.

Furthermore, miRNAs and TFs are capable to alter each other's expression which results in difficulties for ascertaining the effect either one has on the target gene (TG) expression. The Integrated network (Fig. 3) consists of a total of 1396 nodes and edges 17,248 is constructed using Cytoscape from 235 (85 + 150) DE TGs, 24 regulating TFs and 1100 (846 up regulators + 1055 down regulators; 801 are common for both) very high target miRNA. In the network analysis, 14 clusters are achieved. The highest score of the cluster 1 is found to be 5.22 as given in Table 3. The present Gene-TFs-miRNAs regulatory network to find out the potential regulatory and regulated nodes provide detail insight for the gene associations, regulations by relevant TFs, and controlling the miRNAs.

**Table 3:** GO: biological process of Gene of all the clusters.

GOID	GOTerm	Term *P* value	Nr. genes	Associated genes
GO:0032482	Rab protein signal transduction	9.73404E−12	11	[RAB12, RAB13, RAB2B, RAB31, RAB40A, RAB40AL, RAB40C, RAB7A, RAB8B, RAB9B, RNASE4]
GO:0031570	DNA integrity checkpoint	2.18109E−05	9	[AURKA, BTG2, CCND1, CDC5L, CDH1, CDKN1A, EP300, TOP2A, TP53]
GO:0030968	Endoplasmic reticulum unfolded protein response	1.3072E−05	8	[ATF3, BIRC3, CCND1, CXCL8, DDIT3, EP300, PPP1R15A, STC2]
GO:0034620	Cellular response to unfolded protein	3.59987E−05	8	[ATF3, BIRC3, CCND1, CXCL8, DDIT3, EP300, PPP1R15A, STC2]
GO:0042770	Signal transduction in response to DNA damage	4.69977E−05	8	[AURKA, BTG2, CDC5L, CDH1, CDKN1A, EP300, FOXM1, TP53]
GO:0071156	Regulation of cell cycle arrest	1.24845E−05	8	[AURKA, BIRC3, BTG2, CCND1, CDKN1A, EP300, FOXM1, TP53]
GO:0042107	Cytokine metabolic process	5.50804E−05	7	[EGR1, ERRFI1, LTB, STAT3, TICAM1, TNF, ZFP36]
GO:0042089	Cytokine biosynthetic process	5.26779E−05	7	[EGR1, ERRFI1, LTB, STAT3, TICAM1, TNF, ZFP36]
GO:0042035	Regulation of cytokine biosynthetic process	2.84863E−05	7	[EGR1, ERRFI1, LTB, STAT3, TICAM1, TNF, ZFP36]
GO:0043618	Regulation of transcription from RNA polymerase II promoter in response to stress	9.16051E−05	7	[ATF3, CITED2, CREBBP, DDIT3, EGR1, EP300, TP53]
GO:0072395	Signal transduction involved in cell cycle checkpoint	7.42013E−06	7	[AURKA, BTG2, CDC5L, CDH1, CDKN1A, EP300, TP53]
GO:0044774	Mitotic DNA integrity checkpoint	6.56057E−05	7	[AURKA, BTG2, CCND1, CDKN1A, EP300, TOP2A, TP53]
GO:0072401	Signal transduction involved in DNA integrity checkpoint	6.96437E−06	7	[AURKA, BTG2, CDC5L, CDH1, CDKN1A, EP300, TP53]
GO:0072422	Signal transduction involved in DNA damage checkpoint	6.96437E−06	7	[AURKA, BTG2, CDC5L, CDH1, CDKN1A, EP300, TP53]
GO:0006970	Response to osmotic stress	0.000105185	6	[ERRFI1, MAP2K7, RELB, TNF, TSC22D3, TSC22D4]
GO:0048708	Astrocyte differentiation	0.000105185	6	[CLCF1, PTHLH, SMOX, SOX9, STAT3, TNF]
GO:0044783	G1 DNA damage checkpoint	3.63554E−05	6	[AURKA, BTG2, CCND1, CDKN1A, EP300, TP53]
GO:0044819	Mitotic G1/S transition checkpoint	3.41466E−05	6	[AURKA, BTG2, CCND1, CDKN1A, EP300, TP53]
GO:0031571	Mitotic G1 DNA damage checkpoint	3.41466E−05	6	[AURKA, BTG2, CCND1, CDKN1A, EP300, TP53]
GO:0045747	Positive regulation of Notch signaling pathway	0.000106301	5	[CREBBP, ELF3, EP300, SLC35C2, STAT3]
GO:0061614	Pri-miRNA transcription by RNA polymerase II	3.54965E−05	5	[ETS1, PPARG, SOX9, STAT3, TP53]
GO:0070231	T cell apoptotic process	8.59147E−05	5	[CD274, CLCF1, EFNA1, TP53, TSC22D3]
GO:0006984	ER-nucleus signaling pathway	4.22636E−05	5	[ATF3, CXCL8, DDIT3, PPP1R15A, TP53]
GO:0045662	Negative regulation of myoblast differentiation	5.55529E−05	4	[DDIT3, ID3, SOX9, TNF]
GO:0036499	PERK-mediated unfolded protein response	3.16435E−05	4	[ATF3, CXCL8, DDIT3, PPP1R15A]
GO:1990440	Positive regulation of transcription from RNA polymerase II promoter in response to endoplasmic reticulum stress	8.34712E−05	3	[ATF3, DDIT3, TP53]

### Extraction of the cluster network

Using MCODE we extracted total 14 clusters according to the score computed along with nodes and edges, in which only six possesses Gene-TFs-miRNA interactions. In cluster 1 with maximum score, there are mainly Gene–Gene-miRNA interactions. However, gene FOXQ1 is observed in the clusters which is also identified as TF in literature. Few clusters are found to have only gene–gene or gene-miRNA interaction over Gene-TFs or Gene-TFs-miRNA interaction and is given in Supplementary Table S1.

**Figure 4:**
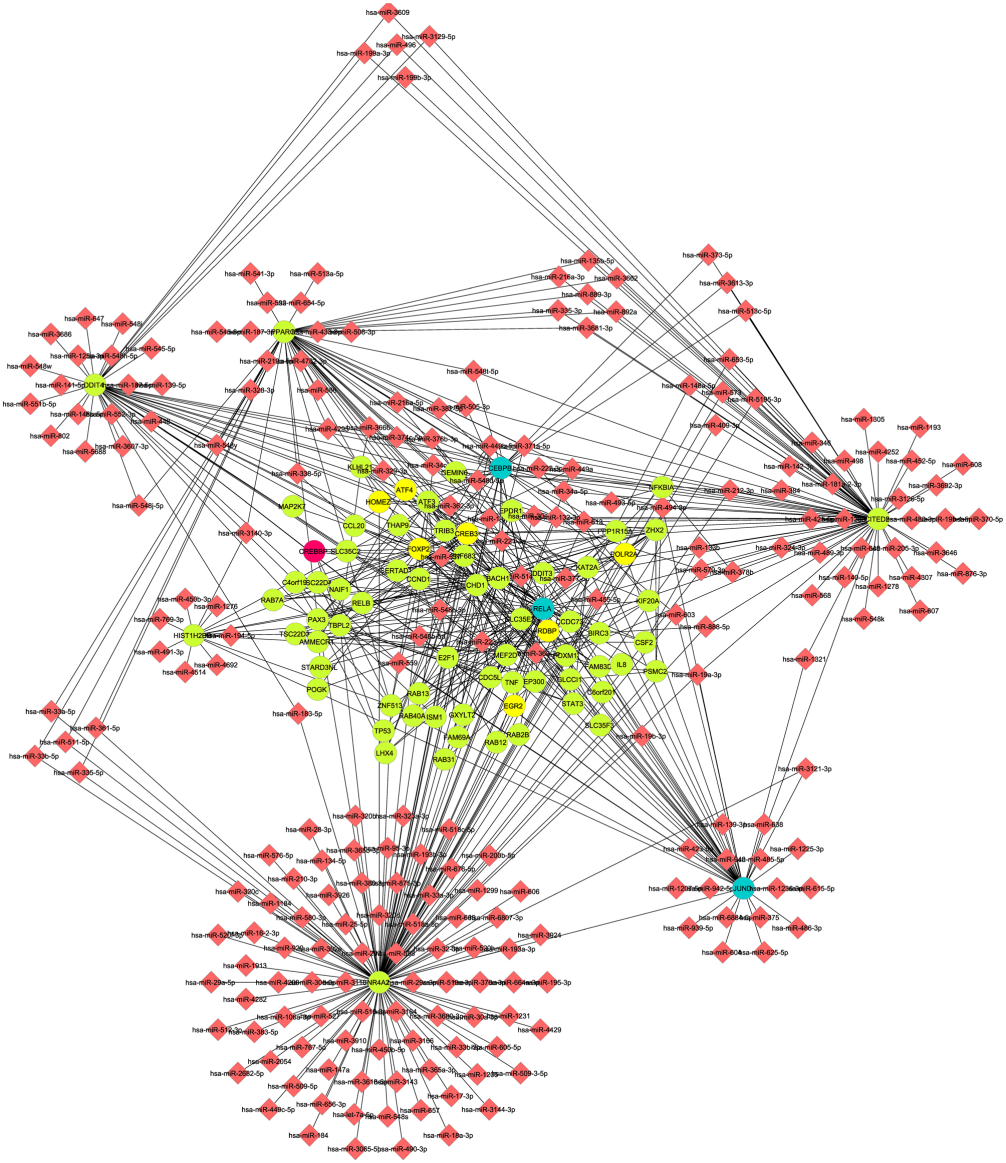
Cluster 3 for Gene–TFs-miRNA regulatory network in Silicosis disease.

From Fig. 4 in cluster 3 (Nodes-312, Edges-621, Score = 3.968), for example, TF JUND is associated with various genes as well as numerous miRNAs altogether. A similar type of network is observed for TFs RELA and PEBPB. In present study, considering the data with crystalline silica exposure of 0 and 800 µg/mL for 6 h exposure, 235 DEGs are enlisted on the basis of FDR set as the cut‑off parameters to screen out 250 significant increases or decreases in gene expression levels. Previous study by Sellamatu et al.^16^ considered a set of exposure (0, 15, 30, 120, 240 µg/cm^2^ for 0–6 h) for their study and on the basis of potential pathway and gene ontology term identified significantly expressed 60 DEGs. Current study emphasise on identification and enlist of the biological pathway and process individually where identified DEGs are involved identifying potential regulating TFs and target miRNA according to the DEGs.

### Functional enrichments analysis

**Figure 5:**
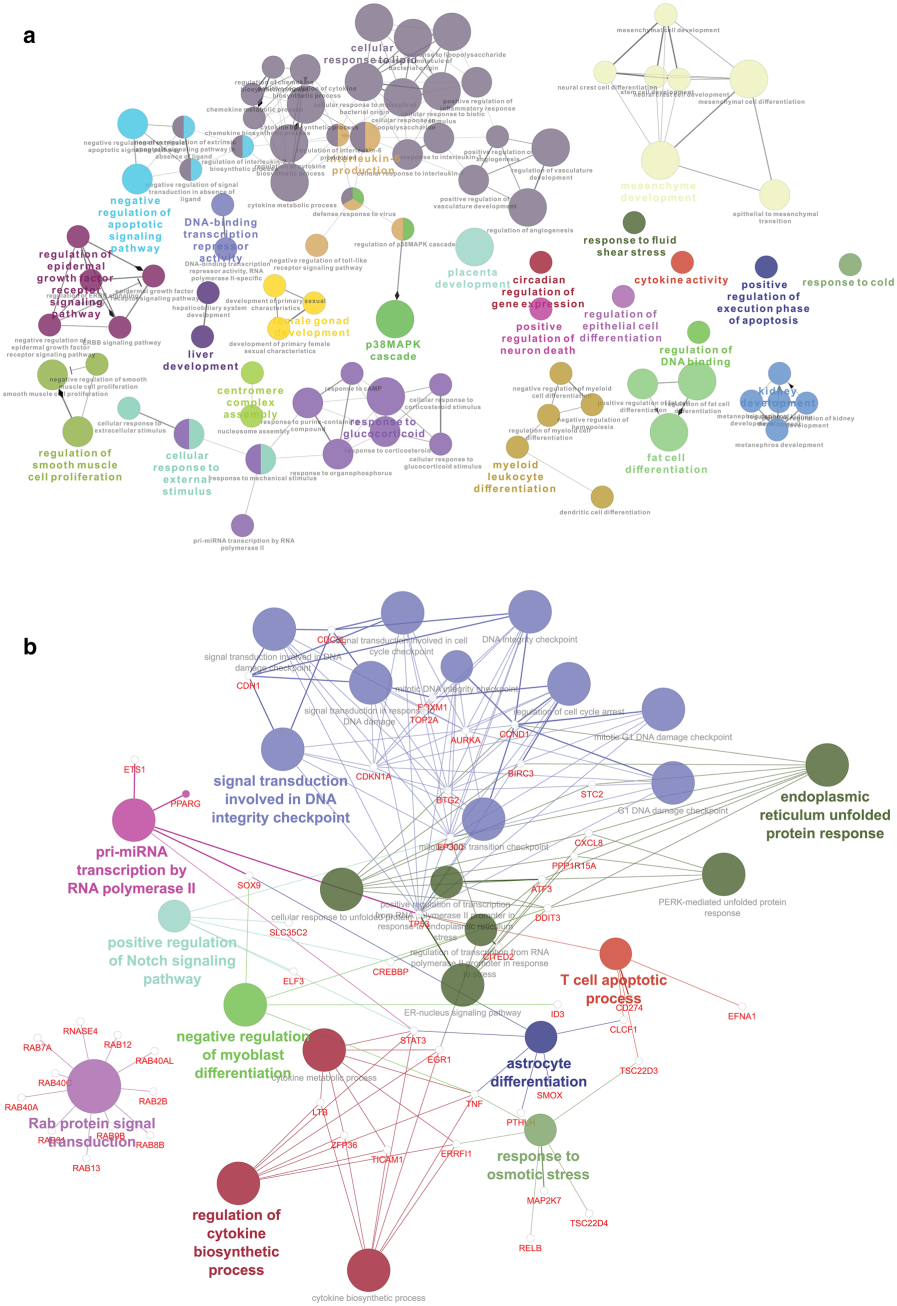
(**a**) GO: Biological process of Gene involved in Silicosis disease. (**b**) GO: Biological process of Gene of all the clusters involved in Silicosis disease.

In present study, biological processes and pathways involoving the 235 DEGs with silica exposure are evaluated and significant biological process with DEGs involved are given in Table 3. A maximum of 11 genes is responsible for Rab proteins signal transduction followed by 9 genes for DNA integrity checkpoint (Fig. 5a). Various researchers reported that overexpression of Rab GTPases have a striking relationship with carcinogenesis and dysregulation of Rab proteins can be linked to the progression of already existent tumors contributing to their malignancy^57,58^.

**Figure 6:**
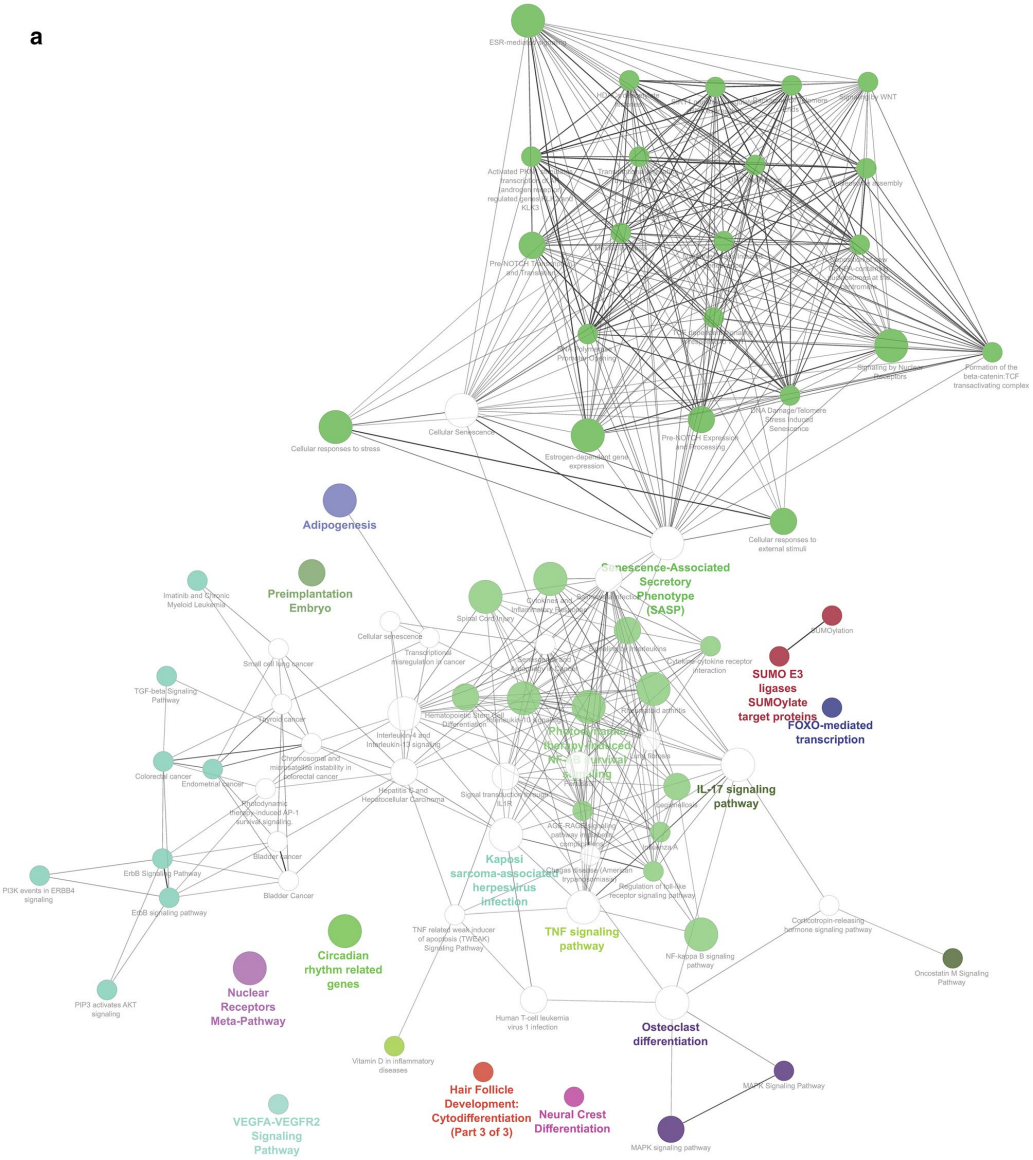

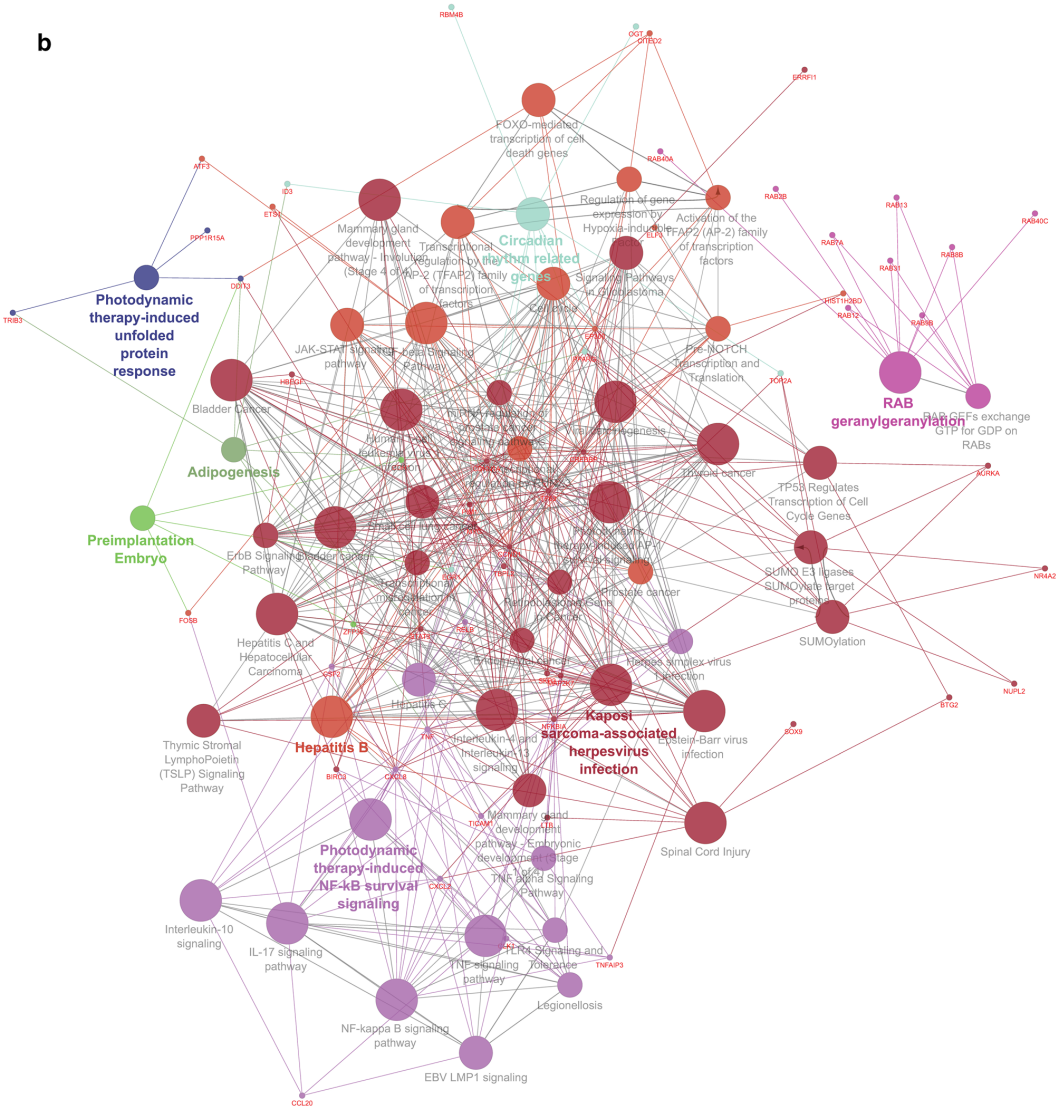
(**a**) GO: Biological pathways of Gene involved in Silicosis disease. (**b**) GO: Biological pathways of Gene from six clusters involved in Silicosis disease.

For Cluster 1, the biological process identified and the genes involved are biological adhesion (TNS3, CD274, and CDH1), biological phase (TOP2A), biological regulation (TOP2A, CD274), cellular process (TN53, IDH1, TOP2A, UBAP1, CD274, and CDH1), immune system process (TN53, CD274), metabolic process (IDH1, and UBAP1) and response to a stimulus (CD274) (Fig. 5b). The biological pathways observed in the study is shown in Fig. 6a,b. Major pathways with higher involvement of genes (numbers given in parenthesis) are Cytokines and Inflammatory Response (6), Rheumatoid arthritis (11), Signaling by Nuclear Receptors (14), NF-kappa B signaling pathway (10), Cellular responses to stress (21), Interleukin-4 and Interleukin-13 signaling (10), Kaposi sarcoma-associated herpes virus infection (13), Interleukin-10 signaling (8), TNF signaling pathway (13), Spinal Cord Injury (13), Circadian rhythm related genes (15), Nuclear Receptors Meta-Pathway (19), IL-17 signaling pathway (16), Cellular Senescence (20), Adipogenesis (11), ESR-mediated signaling (13), Photodynamic therapy-induced NF-kB survival signaling (9), Osteoclast differentiation (11), Senescence-Associated Secretory Phenotype (SASP) (13), and Estrogen-dependent gene expression (13).

Biological pathway analysis of the genes from the six clusters having Gene-TF-miRNA interactions and found the maximum genes are involved in Human T-cell leukemia virus 1 infection as well as Kaposi sarcoma-associated herpesvirus infection (14 nos) followed by Hepatitis B (11 nos) [Supplementary Table (S2)].

### Prediction Small molecule signatures

Table 4 displays the Rank (based on the synergy score), the perturbed molecule (names of the chemical perturbations), Dose, Cell-line, time, the direction of regulation, and the GEO ID from which thesignature is extracted. We next sought to predict the drug combination with the ranked small molecule drug signature list (Table 5). From L1000CDS2 search engine forgene-set search, for differentially expressed genes provided nearly fifty significant combinations. These help infinding reverse and mimic combinations of an input gene expression signature for controlling Silicosis. The maximum scoring small-molecule combinations are CGP-60774 (total 20 combinations) followed by alvocidib (total 15 combinations) and with AZD-7762 (total 24 combinations) as can be seen from Table 5 with a few other drugs having a high probability of success.

**Table 4:** Drug signature predicted at each time point.

Time	Cell-line	Rank	Score	Perturbation	Dose (um)	Time (j)	Cell-line	Rank	Score	Perturbation	Dose (um)
3 h	HME1	1	0.236	CGP-60474	0.12	3	HS578T	10	0.1798	CGP-60474	0.37
		2	0.2303	Alvocidib	3.33			13	0.1798	BMS-387032	1.11
		3	0.2135	CGP-60474	0.04			16	0.1742	CGP-60474	3.33
		4	0.1966	A443654	1.11			25	0.1685	Alvocidib	10
		5	0.1966	Alvocidib	1.11			29	0.1629	PF-431396	10
		6	0.1966	Alvocidib	0.37			31	0.1629	BMS-387032	3.33
		8	0.191	Alvocidib	0.12			25	0.1685	Alvocidib	10
		9	0.1854	PF-562271	10			29	0.1629	PF-431396	10
		11	0.1798	CGP-60474	3.33			44	0.1517	WZ-3105	10
		12	0.1798	CGP-60474	1.11			31	0.1629	BMS-387032	3.33
		17	0.1742	BMS-387032	1.11			34	0.1573	CGP-60474	1.11
		18	0.1742	CGP-60474	0.37			37	0.1573	A443654	3.33
		21	0.1685	AZD-5438	10			44	0.1517	WZ-3105	10
		22	0.1685	BMS-387032	0.37	3	MCF10A	7	0.1966	BMS-387032	3.33
		23	0.1685	BMS-387032	3.33			14	0.1798	Alvocidib	0.37
		24	0.1685	AT-7519	1.11			19	0.1742	CGP-60474	3.33
		30	0.1629	Linifanib	10			26	0.1685	CGP-60474	0.37
		35	0.1573	AZD-7762	3.33			38	0.1573	CGP-60474	0.12
		36	0.1573	Dasatinib	3.33			39	0.1573	CGP-60474	10
		45	0.1517	AT-7519	3.33			46	0.1517	AZD-5438	10
	MDAMB231	15	0.1798	Alvocidib	0.12	6.0	PC3	32	0.1573	Daunorubicin hydrochloride	10.0
		27	0.1685	AT-7519	1.11			33	0.1573	16-HYDROXYTRIPTOLIDE	0.08
		28	0.1685	CGP-60474	0.12			43	0.1517	Triptolide	10.0
		40	0.1573	BMS-387032	1.11	24.0	HA1E	20	0.1685	Geldanamycin	10.0
		41	0.1573	CGP-60474	3.33	6.0	HCC515	50	0.1461	ER 27319 maleate	10.0
		42	0.1573	CGP-60474	1.11						
		47	0.1517	AT-7519	3.33						
		48	0.1517	CGP-60474	10						
		49	0.1517	Alvocidib	3.33

**Table 5:** Small molecule drug signature and combination.

Rank	Score	Combination		Rank	Score	Combination	
1	0.3371	1. CGP-60474	35. AZD-7762	26	0.2865	1. CGP-60474	33. 16-HYDROXYTRIPTOLIDE
2	0.3258	1. CGP-60474	20. Geldanamycin	27	0.2865	5. Alvocidib	35. AZD-7762
3	0.3202	2. Alvocidib	35. AZD-7762	28	0.2865	6. Alvocidib	35. AZD-7762
4	0.3146	2. Alvocidib	20. Geldanamycin	29	0.2865	8. Alvocidib	35. AZD-7762
5	0.3146	3. CGP-60474	20. Geldanamycin	30	0.2865	26. CGP-60474	35. AZD-7762
6	0.3146	3. CGP-60474	35. AZD-7762	31	0.2865	2. Alvocidib	36. Dasatinib
7	0.3146	1. CGP-60474	36. Dasatinib	32	0.2865	7. BMS-387032	36. Dasatinib
8	0.309	7. BMS-387032	35. AZD-7762	33	0.2865	13. BMS-387032	36. Dasatinib
9	0.309	13. BMS-387032	35. AZD-7762	34	0.2865	14. Alvocidib	36. Dasatinib
10	0.3034	14. Alvocidib	35. AZD-7762	35	0.2865	35. AZD-7762	38. CGP-60474
11	0.2978	19. CGP-60474	35. AZD-7762	36	0.2809	1. CGP-60474	2. Alvocidib
12	0.2978	25. Alvocidib	35. AZD-7762	37	0.2809	2. Alvocidib	3. CGP-60474
13	0.2978	28. CGP-60474	35. AZD-7762	38	0.2809	1. CGP-60474	10. CGP-60474
14	0.2978	29. PF-431396	35. AZD-7762	39	0.2809	5. Alvocidib	20. Geldanamycin
15	0.2921	4. A443654	20. Geldanamycin	40	0.2809	7. BMS-387032	20. Geldanamycin
16	0.2921	4. A443654	35. AZD-7762	41	0.2809	10. CGP-60474	20. Geldanamycin
17	0.2921	10. CGP-60474	35. AZD-7762	42	0.2809	11. CGP-60474	20. Geldanamycin
18	0.2921	15. Alvocidib	35. AZD-7762	43	0.2809	12. CGP-60474	20. Geldanamycin
19	0.2921	16. CGP-60474	35. AZD-7762	44	0.2809	20. Geldanamycin	24. AT-7519
20	0.2921	27. AT-7519	35. AZD-7762	45	0.2809	9. PF-562271	35. AZD-7762
21	0.2921	31. BMS-387032	35. AZD-7762	46	0.2809	18. CGP-60474	35. AZD-7762
22	0.2921	3. CGP-60474	36. Dasatinib	47	0.2809	32. Daunorubicin hydrochloride	35. AZD-7762
23	0.2865	6. Alvocidib	20. Geldanamycin	48	0.2809	34. CGP-60474	35. AZD-7762
24	0.2865	8. Alvocidib	20. Geldanamycin	49	0.2809	8. Alvocidib	36. Dasatinib
25	0.2865	9. PF-562271	20. Geldanamycin	50	0.2809	19. CGP-60474	36. Dasatinib

## Conclusions

Our study targets to provide detailed guidance for future fundamental researches along with some key genes, TFs and miRNAs, which are potential biomarkers for silicosis. The approach used in the manuscript allows us to incorporate various data sources into an integrated network, analysis of network parameters in order to find key network elements. Using various data sources, we found the relationships between different molecular components to support our comprehension of how silicosis progresses. In this study, 235 differentially expressed genes (150 down-expressed and 85 up-expressed) are identified as affected by exposure to crystalline silica. These genes are regulated by 24 TFs and 1100 very high target miRNAs. The network between DEGs, TFs and miRNAs are constructed using the various plug-in of Bioconductor, Cytoscape and MCODE to find the associateship of their various aspects. Total of 14 clusters of the network is achieved where only six clusters there is Gene-TFs-miRNA interaction and other eight clusters posess DEGs–miRNAs interactions or DEGs–DEGs–miRNAs interactions The most targeted genes and TFs as well as miRNAs are observed in cluster 3 to cluster 5 in the network analysis that may help in providing a detailed diagnosis of the disease for its cure. Maximum interacted DEGs with miRNA in terms of category are CDK19 (up-regulated) and ARID5B (down-regulated). Maximum interacted DEGs with DEGs and TFs are CEBPG, RELA, BACH1, FOXF1 and CHD1 wheras maximum interacted TFs are NR4A2, CDKN1A, ATF3, ERRFI1, FOSB and EGR1. Functional analysis of the DEGs given that the highest number (11) genes are responsible for Rab proteins signal transduction (Biological Process). Also, Cellular Senescence (20), IL-17 signaling pathway (16) and Signalling by Nuclear Receptors (14) is the dominant Biological Pathway among others. Maximum scoring small-molecule combinations are CGP-60774 (total 20 combinations) followed by alvocidib (total 15 combinations) and with AZD-7762 (total 24 combinations) is found with few other drugs having a high probability of success.

## Supplementary information

Supplementary Information 1.

Supplementary Information 2.
