# Supplementary material for: Investigation of MicroRNA and transcription factor mediated regulatory network for silicosis using systems biology approach
Source: Sci Rep. 2021 Jan 14;11:1265. doi: 10.1038/s41598-020-77636-4 (PMC7809153; doi:10.1038/s41598-020-77636-4)
Supplement: Supplementary file 2 — Supplementary Information 2. [file 41598_2020_77636_MOESM2_ESM.pdf]

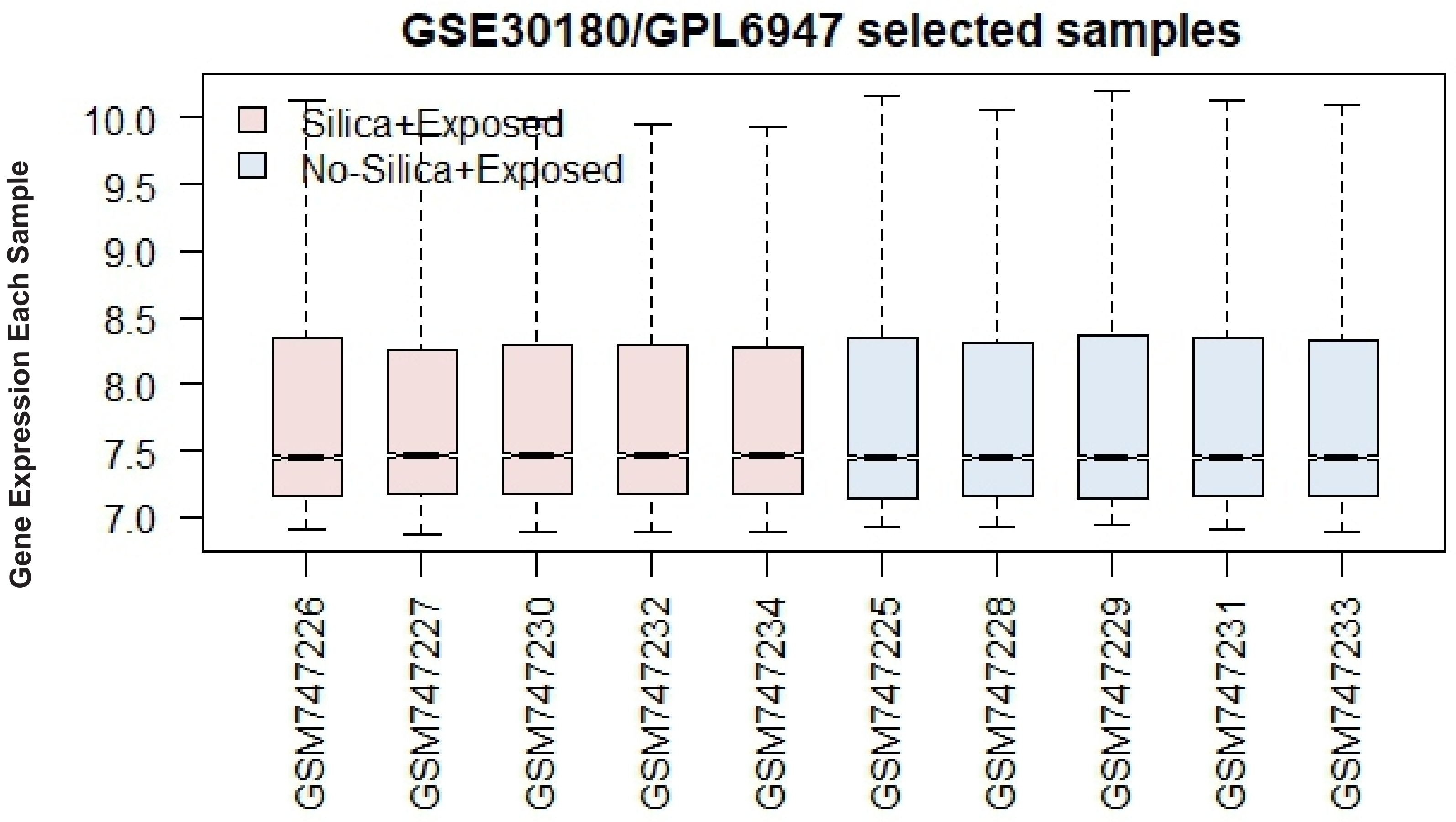

Figure. S1 Distribution values for the differential expression genes

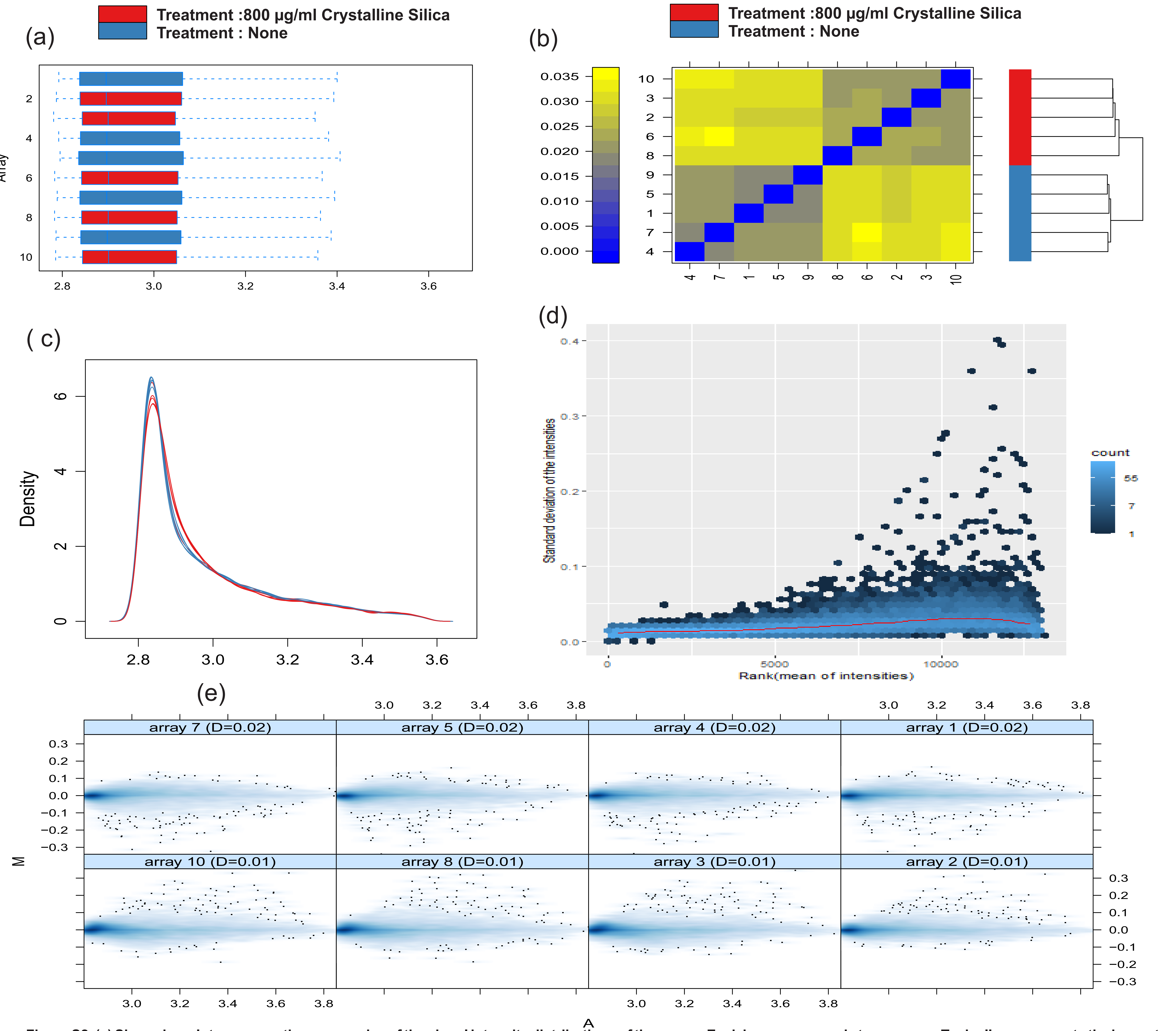

Figure S2 (a) Shows boxplots representing summaries of the signal intensity distributions of the arrays. Each box corresponds to one array. Typically, one expects the boxes to have similar positions and widths. If the distribution of an array is very different from the others, this may indicate an experimental problem. (b) Shows a false color heatmap of the distances between arrays. The color scale is chosen to cover the range of distances encountered in the dataset. Patterns in this plot can indicate clustering of the arrays either because of intended biological or unintended experimental factors (batch effects). (c) shows density estimates (smoothed histograms) of the data. Typically, the distributions of the arrays should have similar shapes and ranges. Arrays whose distributions are very different from the others should be considered for possible problems. (d) Shows a density plot of the standard deviation of the intensities across arrays on the y-axis versus the rank of their mean on the x-axis. The red dots, connected by lines, show the running median of the standard deviation. After normalisation and transformation to a logarithm(-like) scale, one typically expects the red line to be approximately horizontal, that is, show no substantial trend. (e) shows MA plots. M and A are defined as: M = log2(I1) - log2(I2) A = 1/2 (log2(I1)+log2(I2)), where I1 is the intensity of the array studied,and I2 is the intensity of a "pseudo"-array that consists of the median across arrays. Typically, we expect the mass of the distribution in an MA plot to be concentrated along the M = 0 axis, and there should be no trend in M as a function of A. If there is a trend in the lower range of A, this often indicates that the arrays have different background intensities; this may be addressed by background correction. A trend in the upper range of A can indicate saturation of the measurements; in mild cases, this may be addressed by non-linear normalisation (e.g. quantile normalisation).
